# Double Pneumonia

**Published:** 1881-04-20

**Authors:** E. P. Townsend


### DOUBLE PNEUMONIA.

BY E. P. TOWNSEND, M. D.

In view of the recent articles by Dr. Hiram Corson, in the *Medical and Surgical Reporter*, also one in the February number of this journal, by Dr. Ezra Michener, and a reprint in the present number of one by Professor S. D. Gross, upon the subject of "Bloodletting as a Remedy," each of whom refers to pneumonia as one of the diseases whose fatality has increased since bloodletting has gone out of fashion, I have thought it advisable to report the clinical notes of a case occurring recently in my practice. The notes were taken at the bed-side, and carefully taken with a view to publication, since the practice is such as I have followed for upwards of fifteen years, with excellent success; in fact with such success that I believe that cases of this kind occurring in previously healthy individuals, and seen in the early stages, should be almost invariably saved. The patient was Mr. Henry O., aged twenty years. I have known him since he was five years old. He has had no previous sickness of a serious character; no hereditary taint; weight about one hundred and forty, height five feet four; is noted for his endurance in athletic sports; a tinsmith by trade.

On the 8th of January, he was on the river skating most of the afternoon; left the ice at Burlington quite warm from the exercise, and walked home, a distance of three miles. On the 9th he found he had contracted a severe cold, but again went to the river and skated for some time. On the 10th and 11th he remained in the house and used some domestic remedies. I was called to see him at 8 o'clock, on the evening of the 11th, and found that he had just had a severe chill. His breathing was thirty-six to the minute, respirations shallow, a hectic flush on each cheek; in the bowl at the bedside was considerable rusty and blood-streaked sputa; the pulse was 120 and the thermometer registered 104° in the axilla. There was very little thoracic movement in respiration, the breathing being diaphragmatic. Auscultation revealed crepitant rales in lower lobes anteriorily, and in the whole posterior surface of both lungs; skin dry and hot. I ordered tinct. verat viride, gtts. iij every hour until the pulse was reduced to 70; the whole anterior surface of the chest to be covered with silk oil cloth, next the skin. Saw the patient again at 12 m., and found the pulse 108, temperature 102°. I remained with him and continued the treatment until 4 a. m., when the pulse had dropped to 80; temperature 101°; skin moist; respirations twenty-four; expectoration more free and containing much more blood. The veratrum was ordered to be given every two hours if the pulse was above eighty, and I left him in charge of his mother.

12th, 8 a. m.—Nurse and patient slept from 5 until 8, and no medicine given, the skin was again dry, pulse 104, temperature 103°; no veratrum had been given. 3 p. m. Ordered it to be given again once in two hours until I returned, unless the pulse was reduced to 70 and the patient prostrated, or vomiting ensued. At 12 m., pulse 100; the patient sweating freely, breathing more easy and the expectoration more profuse and more blood. The patient in disobedience of orders, had insisted upon talking a great deal. At 6 p. m., pulse 70, temperature 98°; patient perspiring freely; mucous rales over lower anterior lobes of each lung.

13th, 8 a. m.—Patient perspired freely all night; veratrum had not been used but once; pulse 90, temperature 98°; ordered

℞ Ammon carb.......................................... ʒj.

Muc. acacia.......................................... ℥iv.

Tr. aconite.......................................... gtts. xvj

M. f. Emul. Sig. Teaspoonful to be given every two hours; veratrum discontinued.

12 m.—Pulse 100, temperature 101.5, respiration 30; moisture on forehead only; discontinued the ammonia, and re-ordered the veratrum until the pulse was reduced to 70 again. At 6 p. m., veratrum again discontinued and ammonia substituted.

14th, 8 a. m.—Pulse 100, soft and feeble, perspiration and expectoration profuse; continued ammonia. In addition, ordered the patient ʒij of brandy in ℥ij of milk every four hours, to be alternated with beef tea; the temperature had dropped to 98.5°. Continued this treatment throughout the day and night.

15th, 8 a. m.—The sputa is abundant and sanguineous, it also contains lumps of clear jelly-like matter; the face is pale and perspiration very free, especially about the head. Changed the remedy as follows:

℞ Ammon carb.......................................... ʒj.

Muc. acacia.......................................... ℥iij.

M. emulsion, et ad.

Tr. gentian.......................................... ℥j.

Sig. Teaspoonful every two hours. Brandy, milk and beef tea continued.

16th.—Patient convalescing, pulse 90, respiration 24, expectoration less profuse, still glutinous and bloody; treatment continued. There was no further drawback, and the patient left his room on the 20th, and was discharged on the 22d.

I give the above case in full to illustrate the following points. First the patient was of the sthenic type. The disease of acute form, and it had not yet reached the second stage. What kept up the engorgement of the lung? The rapid action of the heart; and evidently the indication was to quiet the heart. How should I do it? I had a choice of means; the lancet, veratrum viride, aconite. Either of them was certain to do it if properly used. Every man works best with the implements he is most familiar with. I was accustomed to using veratrum; I knew it would do the work, and I used it as fearlessly as either of the gentlemen named above would have used the lancet. When it had accomplished its work I ceased using it.

The patient was prostrated, but no more than he would have been with the lancet, and he had all of his blood in his veins, except what he had lost through the lungs. In his weak condition his system was not called upon to manufacture thirty or forty ounces of blood to supply deficiencies. In olden times the advocates of bloodletting claimed that the blood was bad and must be removed, and pointed to the buffy coat as a proof of their theory, but our recent advocates lay no such claim; they bleed to reduce the power of the heart. Then why not reduce the power of the heart with veratrum and save the blood? All the modern talk of change of type of disease is sheer nonsense, and the type of any disease in a patient depends entirely upon the physical condition of that patient when attacked. There is no doubt bloodletting would be just as beneficial when necessary, as it was in former days; but what's the use of losing the volume of blood when the heart can be controlled just as completely without it? It must be borne in mind that Chemistry and Pharmacy have made rapid strides in the last half century. The statement that pneumonia has been more fatal in late years than formerly, may be true, but the fault lies in false teaching and bad judgment.

Professor Flint, not many years ago, taught and advocated throughout the land that pneumonia must be treated with stimulants, and Professor Flint was right, so far as the clinical cases who presented themselves at public institutions, suffering in the second and third stages of the disease, with constitutions previously shattered, by want and exposure, or rum and syphilitic taint, were concerned, but that any country doctor could go to the bedside of a pneumonic patient who was strong and robust but a few hours before, and pour whisky on the already blazing fire that consumed him, is simply rediculous; and yet they did it because Austin Flint ordered whisky in pneumonia.

The proper timing of remedies is also a great element of success. Professor Gross, Hiram Corson or Ezra Michener, would neither of them advise bleeding every man that had pneumonia, simply because he had it. Every physician who knows how to use a fly blister, knows that his patient must be in a certain condition before a blister is available. As well use the lancet or veratrum in the last stage when life is fast ebbing out, as use whisky or ammonia when the whole system is on fire, and the heart beating furiously and forcing the blood into the injured lung in torrents. The lancet, veratrum and aconite have all been maligned because they have been misused and abused by persons unfamiliar with the tools they were using, and the disease they are trying to combat.

One great cause of the increased fatality of inflammatory or acute diseases at the present day, is the pusillanimity of medical men. It is fashionable to be skeptical about the effects of remedies, to preach of hygienic measures, of the self-limitation of disease, of time and rose water; but when a physician is called to the bedside of a patient, suffering with an acute inflammatory affection, Professor Gross will agree with me it is the time to roll up his sleeves and fight for the life of his patient.—*Country Practitioner.*

